# Effects of Fire Suppression Agents and Weathering in the Analysis of Fire Debris by HS-MS eNose

**DOI:** 10.3390/s18061933

**Published:** 2018-06-14

**Authors:** Barbara Falatová, Marta Ferreiro-González, Carlos Martín-Alberca, Danica Kačíková, Štefan Galla, Miguel Palma, Carmelo G. Barroso

**Affiliations:** 1 Department of Fire Protection, Faculty of Wood Sciences and Technology, Technical University in Zvolen, ul.T. G. Masaryka 2117/24, 960 53 Zvolen, Slovakia; xfalatova@is.tuzvo.sk (B.F.); danica.kacikova@tuzvo.sk (D.K.); 2 Department of Analytical Chemistry, Faculty of Sciences, University of Cadiz, Agrifood Campus of International Excellence (ceiA3), IVAGRO, P.O. Box 40, 11510 Puerto Real, Cadiz, Spain; miguel.palma@uca.es (M.P.); carmelo.garcia@uca.es (C.G.B.); 3 Department of Analytical Chemistry, Physical Chemistry and Chemical Engineering, Edificio Polivalente de Química, and University Institute of Research in Police Sciences (IUICP), 28871 Alcalá de Henares, Madrid, Spain; carlos.martina@uah.es; 4 Fire Research Institute of the Ministry of Interior, Rožňavská 11, 831 04 Bratislava, Slovakia; stefan.galla@minv.sk

**Keywords:** chemometrics, headspace-mass spectrometry electronic nose, fire debris, fire suppression agents, gasoline, ignitable liquid residues, weathering

## Abstract

In arson attacks the detection of ignitable liquid residues (ILRs) at fire scenes provides key evidence since ignitable liquids, such as gasoline, are commonly used to initiate the fire. In most forensic laboratories gas chromatography-mass spectrometry is employed for the analysis of ILRs. When a fire occurs, suppression agents are used to extinguish the fire and, before the scene is investigated, the samples at the scene are subjected to a variety of processes such as weathering, which can significantly modify the chemical composition and thus lead to erroneous conclusions. In order to avoid this possibility, the application of chemometric tools that help the analyst to extract useful information from data is very advantageous. The study described here concerned the application of a headspace-mass spectrometry electronic nose (HS-MS eNose) combined with chemometric tools to determine the presence/absence of gasoline in weathered fire debris samples. The effect of applying two suppression agents (Cafoam Aquafoam AF-6 and Pyro-chem PK-80 Powder) and delays in the sampling time (from 0 to 48 h) were studied. It was found that, although the suppression systems affect the mass spectra, the HS-MS eNose in combination with suitable pattern recognition chemometric tools, such as linear discriminant analysis, is able to identify the presence of gasoline in any of the studied situations (100% correct classification).

## 1. Introduction

In many criminal activities that lead to fire, such as arson or bomb attacks, ignitable liquids (ILs) such as gasoline or diesel are commonly used as accelerants. After a fire occurs, an investigation must be carried out and this includes the observation and collection of evidence at the scene. Samples collected from fire scenes are then analyzed in a laboratory to determine the presence/absence of ignitable liquid residues (ILRs). The detection of an accelerant at the fire scene can be the difference between classifying a fire as accidental or as arson [1]. Arson is an illegal activity and fire investigation is therefore considered as a forensic science that covers knowledge from various fields [2]. Gasoline is the most commonly identified accelerant reported by American forensic laboratories since it easy to obtain and ignite [3].

Since 1990 The American Society for Testing and Materials (ASTM) has updated and published standard methods for fire debris analysis and created the ignitable liquids (ILs) classification consisting of eight classes of fuels with precise descriptions [4]. Currently, ASTM E1618 describes the standard test method for the identification ILRs in Extracts from Fire Debris Samples by gas chromatography-mass spectrometry (GC-MS) [5]. This methodology is the most widely used in forensic laboratories around the globe. However, during the last decade, significant improvements to this methodology and new alternative approaches have been reported in the literature in order to overcome some of the drawbacks associated this methodology and to address the new challenges faced by fire debris examiners [6]. In this sense, Borusiewicz declared that this methodology is subject to human interpretation since it is based on the evaluation of the total ion chromatogram (TIC) or extracted ion chromatogram (EIC) of the major compounds by visual pattern comparison to a reference database [7]. In addition, this interpretation procedure is time-consuming, does not allow automation and the interpretation of the results becomes more complicated when samples are not a neat IL but collected from fire debris and contain ILRs.

Moreover, the identification of ILs or ILRs is a challenge for fire debris analysts for many other reasons. Firstly, the destructive nature of a fire, together with the high temperatures, destroys and causes evaporation of the accelerant. Furthermore, fire debris samples are usually taken hours after the fire has been extinguished and during this delay they might be subjected to additional chemical modification processes [8,9,10]. All of these phenomena can modify the chemical composition of residues and this may result in a modification of the resulting analytical signal.

The weathering process introduces different IL profiles and leads to the disappearance of some volatile organic compounds (VOCs). As far as gasoline is concerned, lighter VOCs are the main components affected by weathering. There is a specific database of different states of weathered samples that may be useful in pattern recognition [6].

The classification of post-burn samples is complicated owing to the presence of VOCs resulting from substrate background products and combustion or pyrolysis products [11,12].

While some of these phenomena have been studied previously [6], others remain unknown. In some cases, the analysis may be hindered when pyrolysis products contain target compounds determined in one of the IL classes [13]. In addition, chromatograms contain peaks due to ignitable liquid target compounds that are partial or complete [14] and their ratios with respect to substrate pyrolysis vary as they are affected by the temperature, volume of IL and the nature of the substrate [15]. It is important that fire debris analysts are aware of all of these processes in order to draw a reliable conclusion.

During fire intervention, fire suppression agents (FSAs) can also contribute to the modification of products found in fire debris. FSAs can have two effects, namely diminishing the amount of ILR recovered and contributing to the presence of interfering products [1]. Some authors have stated that the use of powder or foam as extinguishing agents produces different effects when compared to the use of water alone [1]. In this sense, Knowlton [16] focused on the effect of aqueous film-forming foams (AFFFs) on the TIC pattern. According to the chromatograms obtained, Knowlton affirmed that compounds change during pyrolysis because lighter hydrocarbon fractions are preserved by the presence of foams. Conversely, other authors concluded that the use of FSAs or compressed air foam did not significantly interfere in the identification of hydrocarbon fuels in fire debris [17,18,19]. There are numerous different types of foams and powders that may affect the conclusions in different ways. For instance, bacteria are added to some fire suppression foams in order to serve in fire scene decontamination and remediation [6]. Further investigations are required into the effects that different FSAs have on the identification of ILs as this is an unresolved issue.

In order to avoid misunderstandings and incorrect conclusions, chemometric tools provide an advantageous approach for the analysis of fire debris. Such tools are becoming more popular in many fields. As highlighted by different studies [6], multivariate statistical methods like linear discriminant analysis (LDA), principal component analysis (PCA), hierarchical cluster analysis (HCA), and soft independent modelling of class analogies (SIMCA) have been applied to chromatographic data from both unburned and post-burned ignitable liquid samples [20,21] and artificial neural networks (ANN) have been applied to gasoline classification in conjunction with near-infrared data [22]. Tan et al. [20] stated that PCA and SIMCA could be successfully applied to classify accelerants after GC-MS. 

The total ion spectrum (TIS) provides an alternative approach for data analysis [13]. The TIS is identical to an average mass spectrum covering the complete chromatographic range. As the TIS contains sufficient information, the electron ionization TISs were successfully used for the accurate identification and classification of ILs in combination with suitable chemometric tools [13,23]. These results are consistent with data obtained in previous studies [6,10,20,23,24,25,26,27] in which the ability of multivariate statistical analysis and chemometrics to discriminate fuel-enriched samples has been demonstrated.

In recent years, new complementary or alternative methodologies to the commonly used methods for fire debris analysis have been proposed, for instance, the headspace-mass spectrometry electronic nose (HS-MS eNose). The HS-MS eNose is a non-separative technique since it gives specific responses to entire volatile compounds in a way similar to human noses without prior separation. For this reason, the eNose provides an overall fingerprint (the total ion mass spectrum or TIMS) of the odor/volatile profile characteristics of each sample. This spectrum is equivalent to the TIS. This HS-MS eNose employs a quadrupole mass spectrometer in which each fragment ion (*m*/*z* ratio) of the detector acts as a “sensor” and its abundance is equivalent to the sensor signal [28]. Furthermore, this “mass sensor” can provide chemical information about the sample and the signal, combined with appropriate chemometric tools, can be used for identification purposes. The eNose has been successfully applied in various fields together with multivariate statistical analysis [29,30,31]. Ferreiro-González et al. successfully optimized and validated a method for the discrimination of commercial gasoline samples [32], for the discrimination of different neat ILs poured onto different surfaces [33] and for the characterization of petroleum -based products in water samples [34]. This technique has also been successfully applied for thermal desorption of ILRs from carbon strips as an alternative to the use of CS_2_ as solvent [25], as well as for the direct and fast analysis of fire debris without any pre-treatment of the sample [35]. The results were validated by comparison with those obtained by the GC-MS reference method [36].

The HS-MS eNose in conjunction with pattern recognition chemometric tools has proven to be a promising tool for the identification of ILs and ILRs in fire debris. A previous study based on the HS-MS eNose demonstrated that weathering processes affected the chemical fingerprint of neat gasoline samples, particularly during the first six hours [37]. However, this approach has never been applied for the identification of gasoline in weathered fire debris samples nor in samples that include different suppression systems commonly used by firefighters.

For this reason, the goal of the research described here was to study the capacity of the HS-MS eNose in combination with chemometric tools (HCA, PCA and LDA) with the aim of (І) discriminating the presence/absence of gasoline in fire debris samples, specifically a burned carpet, exposed to different delay times (from 0 h until 48 h) and exposed to different suppression agents and (Ⅱ) to study the influence of using two different fire suppression agents (Cafoam Aquafoam AF-6 and Pyro-chem PK-80 Powder) in the analysis of gasoline in fire debris.

## 2. Materials and Methods

### 2.1. Fire Derbis Samples

A carpet, specifically a second-hand rug as a commonly used flooring material and complex matrix, was selected as the substrate for the study. Gasoline purchased from a gas station in Alcalá de Henares, Madrid (Spain), was used as an accelerant. Cafoam Aquafoam AF-6 (0.01%) (AUXQUIMIA, Oviedo, Spain), referred as Cafoam in this study, and Pyro-chem PK-80 Powder (AUXQUIMIA), referred to as Powder in this study, were selected as fire suppression agents (FSA). These FSAs were obtained from the Fire Station in Alcalá de Henares, Madrid (Spain), and used for this study as they are the typical agents used by Spanish fire departments to extinguish fires.

Simulated fire debris samples were prepared by a modification of the procedure Destructive Distillation Method for Burning provided by the Bureau of Forensic Fire and Explosives [38]. In this case, one piece of a substrate (5 × 5 cm^2^) was replaced by one piece of carpet (3 × 3 cm^2^) and six pieces with an approximate size of 3 × 1 cm^2^ deployed around it in a metal can covering the same area as stated in the method in order to take more than one sample at each experiment.

A volume of 0.5 mL of gasoline was added to the carpet surface in the can with a vented lid and sequentially placed above a propane torch. When a smoke appeared the samples were burned for approximately two additional minutes. The can was then removed from the flame and allowed to cool down. After a cooling time of approximately three minutes one of the two fire suppression agents was applied by spraying it onto the carpet surface and the sample was covered and re-lit.

The sampling of fire debris was performed after delayed times in order to simulate weathered samples, i.e., 0, 1, 6, 12, 24 and 48 h after burning. All of the combinations of different variations were used for fire debris preparation. A total of 36 burning experiments (metal cans) were prepared. From each can, two samples standing at opposite sides were selected so that a total of 72 samples were analyzed. Samples were denoted by the substrate code followed by a liquid code, the suppression agent and then the sampling delay times. The samples were labelled according to the following codes: carpet (Ca), gasoline (GAS), Powder (P) and Cafoam (C) and finally the delay time. For instance: Ca + GAS + P _ 1 h represents burned carpet with gasoline and fire suppression Powder with sampling performed 1 h after burning. Each small piece of fire debris was placed directly into an HS-MS eNose vial for direct analysis without any pretreatment of the sample.

### 2.2. HS-MS eNose Spectra Acquisition

Analysis of the samples was performed on an Alpha Moss (Toulouse, France) electronic nose based on a mass detection system consisting of an HS 100 static headspace autosampler and a Kronos quadrupole mass spectrometer. Nitrogen was used as the carrier gas. Samples in 10 mL sealed vials (Agilent Crosslab, Santa Clara, CA, USA) were placed in the autosampler oven and heated. Headspace was taken from the vial by a gas syringe. To avoid condensation, the syringe was heated above the sample temperature (+5 °C) and the sample was subsequently injected into the mass spectrometer. Between each sample injection, the gas syringe was flushed with nitrogen to avoid cross-contamination. The experimental conditions used for the headspace sampler were optimized in another study [35] and consisted of the following conditions: incubation temperature 115 °C, incubation time 10 min, agitation speed 500 rpm, syringe type 5 mL, syringe temperature 125 °C, flushing time 120 s, fill speed 100 μL/s, injection volume 4.5 mL and injection speed 75 μL/s. The total time per sample was 15 min. The components in the headspace of the vials were passed directly into the mass detector without any chromatographic separation or sample pre-treatment. In this way, the resulting TIMS gives a fingerprint of the sample. The electron ionization spectra were recorded in the range 45–200 mass-to-charge ratios (*m*/*z*). The instrument control was achieved using Residual Gas Analysis software and the Alpha Soft 7.01 software package (Alpha Moss).

### 2.3. Data Analysis

Recorded total ion mass spectra from burned samples were arranged in a data matrix named D *m* × *n*, where *m* is the number of fire debris samples and *n* is the number of *m*/*z* intensities in the range 45–200. The intensities of each *m*/*z* were taken as independent variables and standardized by feature scaling. All of the MS data were normalized to the base peak at 100%.

Multivariate statistical analyses represented by HCA, LDA and PCA were performed by using IBM SPSS Statistics 22 software (Armonk, NY, USA).

## 3. Results and Discussion

In a previous study the effect of the weathering phenomenon on the resulting total ion mass spectrum (TIMS) was evaluated by the HS-MS eNose for neat gasoline samples poured onto different materials [37]. The results showed that the resulting spectra changed markedly after six hours of weathering. The average TIMS obtained for all of the burned gasoline samples weathered at different times (from 0 to 48 h), including samples with the two FSAs, are shown in Figure S1. Although some differences in the TIMS can be appreciated, a clear trend was not observed.

**Figure 1:**
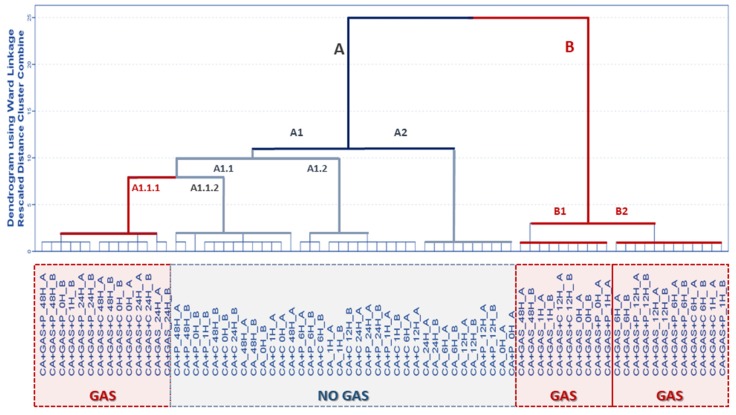
Dendrogram obtained from the HCA for all the fire debris samples using the signal from the HS-MS eNose (45–200 *m*/*z*).

As a visual inspection of the TIMS from the HS-MS eNose is not sufficiently clear to draw any firm conclusion and, as a consequence, chemometric tools that provide automatic and objective data interpretation were applied. Leave-one-out cross-validation was performed. Firstly, the exploratory multivariate analysis technique, namely HCA, was applied with the aim of elucidating any trends for fire debris samples to be grouped according to the usage/non-usage of gasoline and/or type of FSA and/or to the time of sampling. HCA was carried out by using all of the *m*/*z* intensities as independent variables to form groups (D 72 × 156). Squared Euclidean distances were employed to measure distances between the samples. The results from the HCA are graphically represented in a dendrogram in Figure 1. Ward’s hierarchical agglomerative clustering method was used for the HCA.

As can be seen from the dendrogram, fire debris samples are divided into two major clusters—A and B. Cluster A is widely distributed into further subclusters, mostly composed of samples without gasoline. The exception is subcluster A1.1.1, which is formed mainly by samples with gasoline with one of the FSAs (i.e., Cafoam). This subcluster contains 14 out of the total 36 fire debris samples with gasoline and includes 12 out of the 24 burned samples with gasoline and the FSA. Cluster B contains all samples burned with gasoline without FSA (except for those samples with 24 h of weathering, which are included in subcluster A1.1.) and the remaining burned samples with gasoline that also contain Powder or Cafoam. Inspection of the dendrogram suggests that the data from the HS-MS eNose are mainly related to those compounds responsible for the discrimination of the fire debris regarding the use of gasoline, since all of the subclusters include samples with and without gasoline together. However, subcluster A1.1.1, which mainly includes samples with gasoline and FSA, is joined at a shorter distance to subcluster A1.1.2, which includes burned samples without gasoline but including Cafoam or Powder. A trend related to the sampling time was not observed. Therefore, these results suggest that the data from the HS-MS eNose are mainly related to those compounds responsible firstly for the discrimination of the fire debris regarding the use of gasoline and secondly according the type of FSA. A full classification of the samples according to the usage/non-usage of gasoline did not seem to be achieved due to the effect of using FSA.

**Figure 2:**
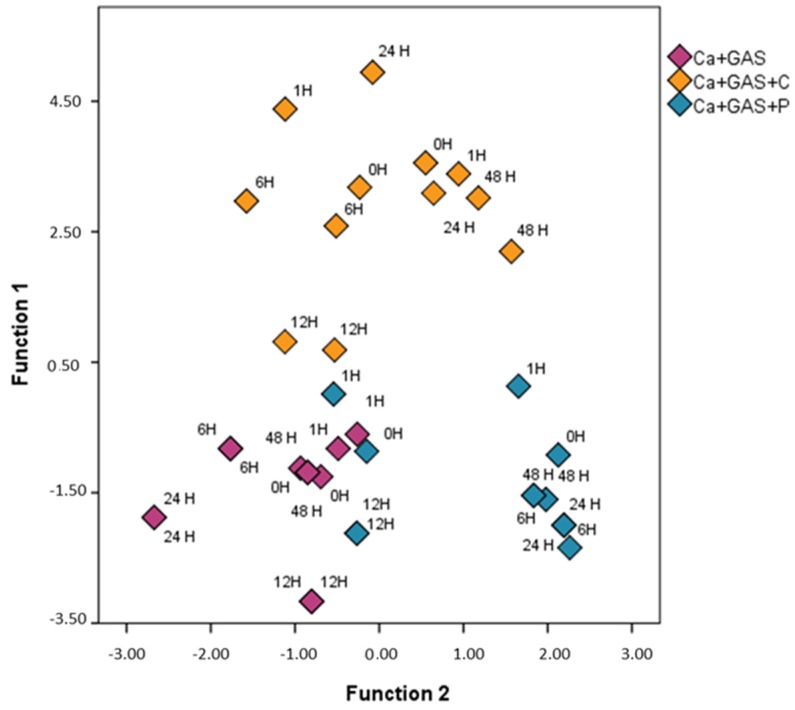
Zone map for the burned samples with gasoline (*n* = 36).

In order to investigate the effect of FSA in greater depth and to maximize the separation between known categories, a supervised chemometric technique, LDA, was applied solely to the dataset of fire debris samples with gasoline. The resulting data matrix was then D 36 × 156. In this case three groups (Ca + GAS, Ca + GAS + C and Ca + GAS + P) were established a priori. A stepwise method was applied in order to identify whether there were specific *m*/*z* values in the TIMS that were more significant than others when discriminating samples according to the use and type of FSA. From this LDA, two canonical discriminant functions that maximize the separation were obtained. The *m*/*z* values selected to develop the discriminant function were: *m*/*z* 51, *m*/*z* 60, *m*/*z* 67, *m*/*z* 87, *m*/*z* 166 and *m*/*z* 190. Since there is not a chromatographic separation, it cannot be guaranteed that the selected *m*/*z* could be related to the different FSAs and/or to other chemical reactions like pyrolysis o weathering processes. The magnitudes of the predictive effect are 0.82 and 0.52, with eigenvalues of 4.59 and 1.09, respectively. A total of 80.6% of cross-validated grouped cases were correctly classified. The territorial map obtained for all the samples burned with gasoline when plotting the first two Fisher’s discriminant functions are shown in Figure 2.

As can be observed, the samples tend to be classified according to the usage/non-usage of FSA. This analysis suggests that the HS-MS eNose also detects some of the FSA chemical compounds in the studied *m*/*z* range and so these may interfere in the identification. As a full discrimination was not achieved, a further study was carried out. In this respect, two additional LDAs were conducted in order to study in greater depth the effect of each FSA used in this study and to try to minimize it. For this purpose, only samples burned with gasoline and samples burned with gasoline and one of the FSAs were analyzed separately, i.e., one for LDA for Ca + GAS and Ca + GAS + C samples and the other for Ca + GAS and Ca + GAS + P samples; the data matrix for both LDAs was thus D 24 × 156. Cross-validation and the stepwise method were again applied to obtain the *m*/*z* values that are more affected by the use of a specific FSA. In both LDAs a full discrimination was achieved. Table 1 includes the Fisher’s linear discrimination functions obtained in each LDA.

As can be observed, *m*/*z* 45, *m*/*z* 49, *m*/*z* 50, *m*/*z* 53, *m*/*z* 60, *m*/*z* 61 and *m*/*z* 78 were selected for the discrimination between samples burned with gasoline with and without Cafoam and *m*/*z* 52, *m*/*z* 55, *m*/*z* 71, *m*/*z* 87, *m*/*z* 119, *m*/*z* 136 and *m*/*z* 198 for the discrimination of samples burned with gasoline with and without Powder. The *m*/*z* values with the highest influence for the discrimination of samples with and without Cafoam were *m*/*z* 49 and *m*/*z* 50, while in the case of samples with and without Powder the values were *m*/*z* 71 and *m*/*z* 198. The *m*/*z* values selected to develop these two discriminant functions were discarded for further analyses, since these *m*/*z* are related to the presence of either of the two FSAs, and so they could interfere in the identification of gasoline in fire debris. Therefore, after removing the information mainly related to the FSA in the samples, the next step was to evaluate the detection of gasoline in the fire debris, which is the ultimate goal of the forensic analyst and expert.

**Figure 3:**
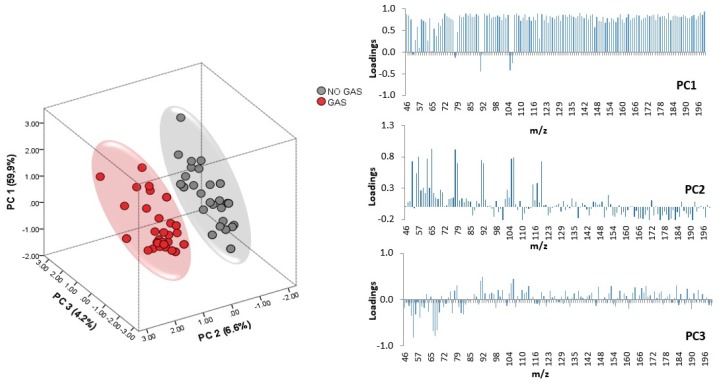
Scores and loadings for the fire debris samples (*n* = 72) in the PC1-PC2-PC3 space.

In an effort to extract more information about the trends suggested by HCA regarding the presence/absence of gasoline, a non-supervised technique, in this case PCA, was applied but using the data matrix (D 72 × 141) without the *m*/*z* intensities selected in the previous LDAs, thus allowing the discrimination between different FSAs used to extinguish the fire. PCA allows the prediction of the correlations between *m*/*z* and samples taking into account the presence of gasoline. Eighteen principal components (PCs) were extracted with eigenvalues greater than 1.00 (cumulative variance 91.79%). The score plot for all the samples according to the first three PCs, which account for 70.77% of the total variance of the original data set, is shown in Figure 3.

As can be observed, PC1, PC2 vs. PC3 allows a full separation of all the fire debris samples according to the presence/absence of gasoline. As illustrated in Figure 3, PC2 seems to be more related to the presence/absence of gasoline although PC1 accounts for a higher variance. The loading values are also displayed in Figure 3. It can be seen that almost all of the *m*/*z* values have a high influence on PC1, while only a few signals show high loadings (values above 0.7) in the case of PC2. Although the contribution of each *m*/*z* is different, the loadings on PC1 are remarkably similar; this is probably due to data normalization procedure.

**Figure 4:**
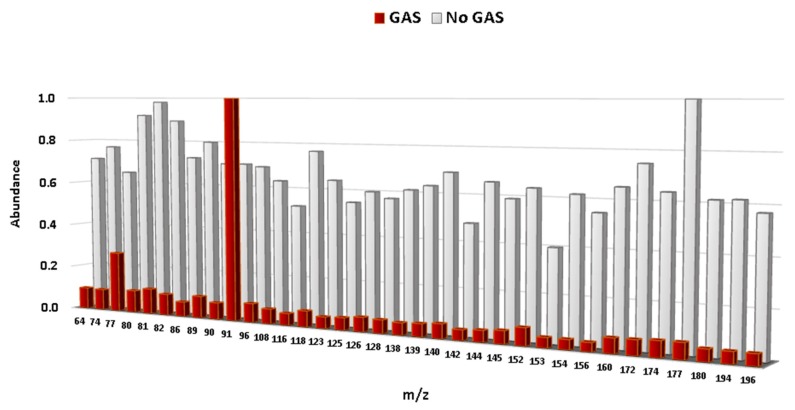
Intensity values of the selected *m*/*z* in the LDA for the burned samples with and without the presence of gasoline.

Based on these results, and with the aim of obtaining a full discrimination of the fire debris samples according to the presence/absence of gasoline, a repeated-analysis stepwise LDA was applied using the whole dataset without *m*/*z* intensities related to the use of the two FSAs (D 72 × 141). Leave-one-out cross-validation was again selected. The resulting discriminant function was able to discriminate successfully all of the samples according to the presence/absence of gasoline (100.0% of cross-validated grouped cases were correctly classified). According to the *p*-value (*p* = 0.000), groups will make predictions that are statistically significant in their accuracy. This indicates that the model is very stable and strong. When only intensity values of the signals (*m*/*z*) selected by the LDA to develop the Fisher linear discriminant functions are displayed, a different profile for burned samples with and without gasoline is obtained (Figure 4). All of the *m*/*z* values were normalized to the base peak at 100%.

It can clearly be seen that the intensity values of the selected *m*/*z* from samples with the presence of gasoline differs significantly from samples without gasoline. Samples burned without gasoline give similar intensities for all signals and do not present any characteristic *m*/*z*. In contrast, samples burned with gasoline show a distinctive signal, *m*/*z* 91, regardless of the use of either FSA. The signal at *m*/*z* 91 is characteristic for the C-2, C-3, and C-4 alkylbenzenes commonly present in gasoline. An additional effect related to the weathering processes was not observed in this study, even though a variable related to weathering (i.e., delayed sampling times) was included in the experiments. These fingerprints can be used for a rapid and objective interpretation of the results.

## 4. Conclusions

The present study was designed to investigate the effect of two FSAs (Cafoam Aquafoam AF-6 and Pyro-chem PK-80 Powder) and delayed sampling times for the identification of gasoline in fire debris samples by HS-MS eNose in conjunction with chemometric tools. The results obtained are significant in at least two major respects. According to the HCA results, the studied FSAs affect the mass spectrum of fire debris containing residual gasoline. Therefore, their chemical composition could interfere with the correct classification of this sort of sample. By applying LDA it was observed that the *m*/*z* with the greatest influence for the discrimination of burned samples with gasoline with Cafoam were *m*/*z* 49 and *m*/*z* 50 and for burned gasoline samples with Powder they were *m*/*z* 71 and *m*/*z* 198. According to this, attention should be paid to the presence of FSAs in other ILs. Additionally, an effect regarding the sampling time (at least until 48 h after extinction and under the experimented conditions) was not observed in this study Finally, HS-MS eNose together with LDA allowed a full discrimination of the samples according to the presence/absence of gasoline regardless of the delay time prior to sampling and the FSA used.

The results obtained demonstrate the importance of considering not only the samples burned with gasoline, but also burned samples that contained FSAs or samples that were not taken immediately but after different times in order to simulate the most reliable scenario.

The results of this research support the idea that the HS-MS eNose can be considered as an alternative analytical tool for fire debris analysis. Further research should be undertaken to explore other aspects of HS-MS eNose to increase its potential for use in fire debris analysis.

## Figures and Tables

**Table 1:** Fisher’s linear discrimination functions obtained in the LDAs for samples with gasoline with/without Powder and samples with gasoline with/without Cafoam.

Classification Function Coefficients					
*m*/*z*	CA + GAS	CA + GAS + P	*m*/*z*	CA + GAS	CA + GAS + C
52	85.550	−165.461	45	450.901	1198.587
55	−156.804	410.264	49	−1025.647	−2630.792
71	675.315	−1538.019	50	822.764	2089.187
87	−145.246	670.559	53	−257.375	−695.097
119	−77.105	496.459	60	−521.821	−1409.675
136	−367.262	365.750	61	114.026	411.485
198	−573.422	1759.694	78	−274.823	−783.013
Constant	−15.090	−39.274	Constant	−44.589	−258.634

## Supplementary Materials

The following are available online at http://www.mdpi.com/1424-8220/18/6/1933/s1, Figure S1: Average total ion mass spectrum for all the fire debris samples burned with gasoline with/without Cafoam or Powder (*n* = 18). A reduced range (*m*/*z* 45–157) is displayed for a better visualization.
