# Supplementary material for: Effects of Fire Suppression Agents and Weathering in the Analysis of Fire Debris by HS-MS eNose
Source: Sensors (Basel). 2018 Jun 14;18(6):1933. doi: 10.3390/s18061933 (PMC6021975; doi:10.3390/s18061933)
Supplement: Supplementary file 1 [file sensors-18-01933-s001.pdf]

Supplementary Materials

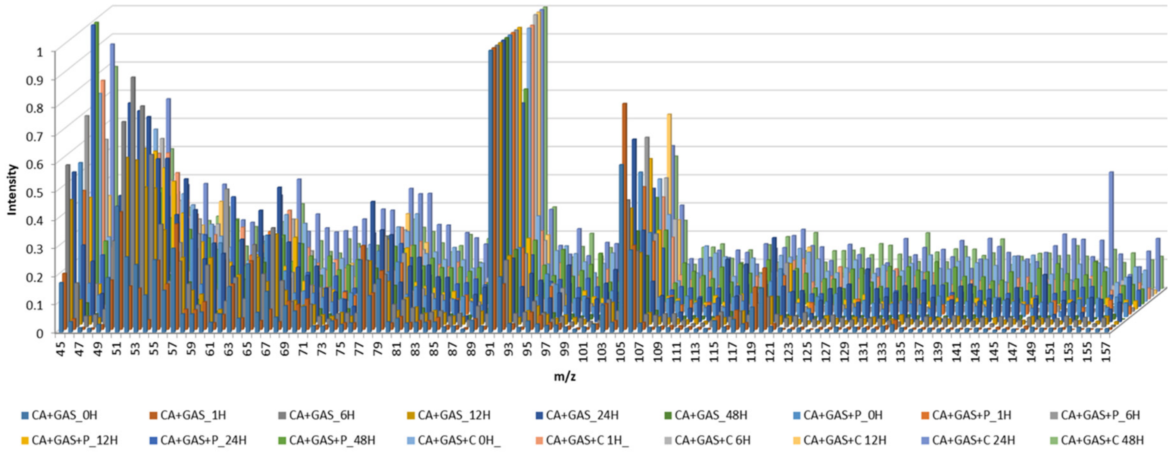

Figure S1. Average total ion mass spectrum for all the fire debris samples burned with gasoline with/without Cafoam or Powder (n = 18). A reduced range (45–157 m/z) is displayed for a better visualization.
